# Sex chromosomes in mitotic and polytene tissues of *Anastrepha fraterculus* (Diptera, Tephritidae) from Argentina: a review

**DOI:** 10.3897/zookeys.540.6058

**Published:** 2015-11-26

**Authors:** María Cecilia Giardini, Fabián H. Milla, Silvia Lanzavecchia, Mariela Nieves, Jorge L. Cladera

**Affiliations:** 1 Laboratorio de Genética de Insectos de Importancia Económica, Instituto de Genética ‘Ewald A. Favret’, CICVyA, Instituto Nacional de Tecnología Agropecuaria (INTA), Hurlingham, Buenos Aires, Argentina; 2 Grupo de Investigación en Biología Evolutiva, Departamento de Ecología, Genética y Evolución, IEGEBA-Facultad de Ciencias Exactas y Naturales, Universidad de Buenos Aires, Buenos Aires, Argentina

**Keywords:** Cytogenetics, karyotype variants, South American fruit fly, heterochromatin, centromeres, ribosomal genes

## Abstract

Cytogenetics, which is considered a fundamental tool to understand basic genetic and genomic issues of species, has greatly contributed to the description of polymorphisms both at inter- and intra-specific level. In fact, cytogenetics was one of the first approaches used to propose *Anastrepha fraterculus* (Diptera: Tephritidae) as a complex of cryptic species. Different morphological variants of sex chromosomes have been reported among Argentinean populations of *Anastrepha fraterculus*. However, since this high structural variability in sex chromosomes does not pose a reproductive barrier, their role in speciation is yet to be unveiled. This review provides an update on general aspects of cytogenetics in Argentinean *Anastrepha fraterculus* populations, focused on the prevalence of X-Y arrangements.

## General background

Cytogenetic studies have provided significant information about intra- and inter-species genetic variation (Sumner 2003). Cytogenetic studies focus mainly on sex chromosomes, which present unusual features relative to autosomes (Traut et al. 2008). In organisms with the classical X-Y systems of sex determination, Y chromosomes lack genetic recombination, are male limited, and show an abundance of genetically inert heterochromatic DNA containing few functional genes, whereas X chromosomes also show sex-biased transmission, and are hemizygous in the heterogametic sex (Kaiser and Bachtrog 2010). In particular, in some insect species, sex chromosomes show high structural variability (see Traut 1999, Kaiser and Bachtrog 2010, Palacios-Gimenez et al. 2013). In the genus *Anastrepha*, Solferini and Morgante (1987), Selivon et al. (2005a), Goday et al. (2006) and Garcia-Martinez et al. (2009) compared different species and reported specific sex-chromosome banding patterns. In addition, they described variability in the length, number, size, and position of heterochromatic blocks in ‘the South American fruit fly’, *Anastrepha fraterculus* Wiedemann (Diptera: Tephritidae). This species is distributed from southern United States to Argentina (Salles 1995, Steck 1999) and constitutes an economically important pest. Currently, it is considered a complex of cryptic species (for reviews and references, cf. Selivon et al. 2004, 2005a, Cáceres et al. 2009, Hernández-Ortiz et al. 2012, Cladera et al. 2014). Multivariate morphological studies including samples from different regions of the American continent have characterized seven distinct morphotypes (Hernández-Ortiz et al. 2012). Studies based on genetic differentiation, karyology, morphology, reproductive compatibilities combined with bionomic parameters, eggshell morphology and some aspects of early embryogenesis of samples from northern and southern Brazil have identified at least four entities of the *Anastrepha fraterculus* complex: *Anastrepha* sp.1 aff. *fraterculus*, *Anastrepha* sp. 2 aff. *fraterculus*, *Anastrepha* sp. 3 aff. *fraterculus*, and *Anastrepha* sp.4 aff. *fraterculus*. The first three entities have been reported in different regions of Brazil, whereas *Anastrepha* sp. 4 aff. *fraterculus* has been described in Guayaquil, Ecuador (Selivon and Perondini 1997, 1998, Selivon et al. 1997, 1999, 2004, 2005a, 2005b, Goday et al. 2006).

Reproductive incompatibilities between *Anastrepha* sp.1 aff. *fraterculus* and *Anastrepha* sp. 2 aff. *fraterculus* living in sympatry were first described by Selivon et al. (1999, 2005a). Later, Vera et al. (2006) showed pre-mating isolation between flies from Peru and Argentina, Brazil and Colombia, as well as between flies from Piracicaba (São Paulo, Brazil) and Argentina. Cáceres et al. (2009) found that hybrids between strains from Peru and Argentina carried the expected mix of sex chromosome cytotypes, but presented sex ratio distortion and high rates of sterility or inviability. High levels of mating isolation have also been reported among Mexican, Peruvian and the Brazilian-1 morphotypes (Rull et al. 2013). Reproductive isolation between the four morphotypes of *Anastrepha fraterculus* complex and flies from “the Andean morphotype” were also found by Devescovi et al. (2014). These and other factors analyzed by the authors are indicative of incipient speciation, providing a strong evidence for a taxonomic revision of this species complex (Selivon et al. 2005a, Cáceres et al. 2009).

Polytene chromosome analysis and the availability of polytene maps of different genera of the family Tephritidae, as *Ceratitis* (Zacharopoulou 1990), *Bactrocera* (Mavragani-Tsipidou et al. 1992, Augustinos et al. 2014), *Dacus* (Drosopoulou et al. 2011), *Rhagoletis* (Kounatidis et al. 2008) and *Anastrepha* (Garcia-Martinez et al. 2009), have allowed identifying differences between closely related species. Moreover, several groups of cryptic species were initially identified using sequences of the polytene chromosomes as genetic markers and later confirmed by molecular markers studies (Ramirez and Dessen 2000).

## Karyotype and sex chromosome configurations

**Figure 1.**
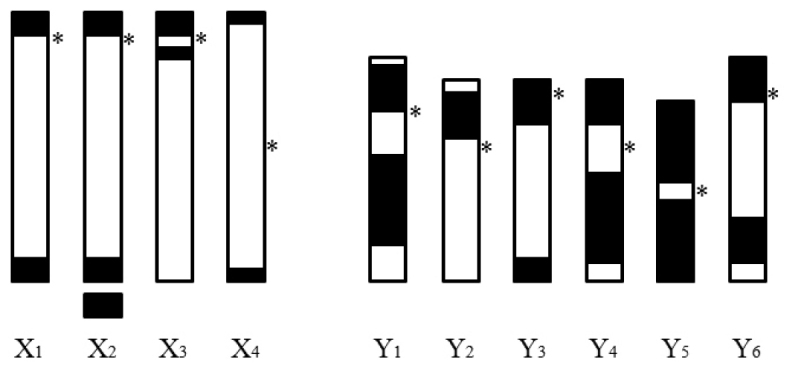
C-Band Ideogram. Sex chromosomes configurations of *Anastrepha fraterculus* found in Argentina (redrawn from Basso 2003). * Position of centromeres in each chromosome.

Karyological studies performed in wild populations of *Anastrepha fraterculus* from Argentina have shown structural variability in the sex chromosomes. Lifschitz et al. (1999) described an acrocentric X chromosome and a small submetacentric Y chromosome (see also Basso and Manso 1998, Basso et al. 2003). Lifschitz et al. (1999) and Manso and Basso (1999) also reported four morphological variants of the X chromosome (named X_1_, X_2_, X_3_ and X_4_) and six variants of the Y chromosome (named Y_1_, Y_2_…Y_6_) at low frequency (Figure 1). Basso and Manso (1998) and Basso et al. (2003) also studied the viability and survival of individuals with different karyotype configurations under laboratory conditions, and showed that the cytogenetic differences found among these Argentinean populations do not represent evidence of reproductively separate species, but seem to be examples of intra-species chromosome polymorphisms.

In an experiment under field cage conditions Petit Marty et al. (2004a, 2004b) confronted *Anastrepha fraterculus* flies from extreme regions (NOA and NEA) inside Argentina and compared the frequency of homotypic and heterotypic crosses. No evidence of sexual incompatibility was found, either pre-zygotic (Petit Marty et al. 2004a) or post-zygotic (Petit Marty et al. 2004b). These studies confirmed the presence of a single *Anastrepha fraterculus* biological entity in Argentina.

**Figure 2.**
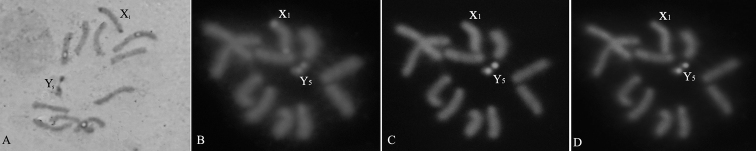
The most frequent karyotype of *Anastrepha fraterculus* found in Argentina. Mitotic chromosome preparations from third instar larvae of *Anastrepha fraterculus* male. **A** C-Bands **B** DAPI stain **C** CMA stain **D** Merged DAPI/CMA images.

After a revision of *Anastrepha fraterculus*´ chromosomes studies we concluded that the most frequent karyotype found in Argentina consists in five pairs of acrocentric autosomes, a submetacentric X chromosome (named X_1_, Lifschitz et al. (1999) (CI average: 31.23) and a metacentric Y chromosome (named Y_5_, Basso 2003) smaller than the X chromosome (Figure 2) (preliminary reported in Giardini et al. 2009b). It is important to highlight that the size and the patterns obtained with the different banding techniques for the X chromosome are the same as the one described by Lifschitz et al. (1999) as X_1_. The only difference between them is the position assigned to the centromere and for that reason we kept on the same name. This difference in the centromere position could probably be explained by the lower resolution in the old pictures obtained by Lifschitz et al. (1999). This karyotype also corresponds to the one that was characterized by Selivon et al. (2005a) using samples from the southern Brazil (*Anastrepha* sp.1 aff. *fraterculus*) although without specifying the centromere position.

After C-band staining (Figure 2A), the X_1_ chromosome shows two prominent and different-sized blocks of heterochromatin located at the terminal region, whereas the Y_5_ chromosome also shows two heterochromatin blocks positioned in different arms, one on the proximal end and the other in the sub-median region (Figure 2B). Both chromosomes show DAPI-positive signals in the same position as the heterochromatic blocks (Figure 2B). No CMA-positive band is distinguishable on the X_1_ chromosome (Figure 2C). However, on the Y_5_ chromosome, CMA-positive bands are observed at the same position as C-bands and DAPI-positive bands (preliminary reported in Giardini et al. 2009b). These observations indicate that the sex chromosomes analyzed in these populations of *Anastrepha fraterculus* differ in the nucleotide composition of the heterochromatic regions: the heterochromatic regions on the X_1_ chromosome are AT-rich, whereas those of the Y_5 _chromosome are AT+CG-rich DNA sequences (preliminary reported in Giardini et al. 2009b). These findings are in agreement with those of Goday et al. (2006).

**Figure 3.**
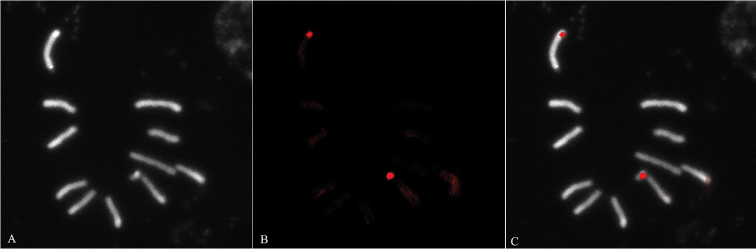
18S rDNA FISH analysis. Mitotic chromosome preparations from third instar larvae of *Anastrepha fraterculus* male. **A** DAPI stain **B** rDNA hybridization signal (autologous probe) **C** Merged images.

*Anastrepha fraterculus* has also been cytogenetically characterized by means of fluorescence *in situ* hybridization (FISH), to locate the ribosomal genes on the chromosome complement. The first studies carried out by Basso and Manso (1998) on cytological preparations of *Anastrepha fraterculus* from Argentina using a heterologous probe from *Drosophila hydei* rDNA described two positive signals at the terminal positions in the X-chromosome. Later, Goday et al. (2006) carried out studies using a *Drosophila melanogaster* probe in a comparative analysis of the *fraterculus* complex using samples from Brazil. In *Anastrepha* sp.1 aff. *fraterculus* individuals, these authors found an rDNA positive signal that co-localized with a DAPI-positive band at a distal position in the X_1_ chromosome, and a second signal at the DAPI/CMA-positive regions of the Y_5_ chromosome. By using a specific probe designed from a region of *Anastrepha fraterculus* 18S rDNA (Figure 3), in our lab we have observed a pattern of signals equivalent to the one previously described by Goday et al. (2006) (preliminary reported in Giardini et al. 2009b). These two last studies confirmed the general tendency observed for the rDNA of reside on the heterochromatic regions of the sex chromosomes other than centromeres (Drosopoulou et al. 2012).

## Chromatin characteristics

**Figure 4.**
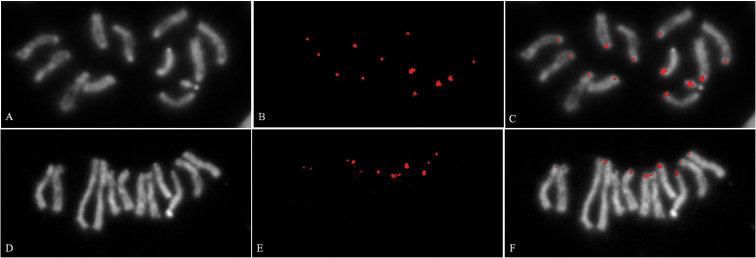
Immunodetection analysis with H3S28ph antibody. Mitotic chromosome preparations of male (**A, B, C**) and female (**D, E, F**) individuals from *Anastrepha fraterculus* **A, D** DAPI stain **B, E** anti-H3S28ph hybridization signal **C, F** Merged images. Arrow heads indicate sex chromosome position.

As a first attempt to study histone modifications in *Anastrepha fraterculus* chromosomes, we performed immunodetection assays with specific antibodies in mitotic preparations of Argentinean *Anastrepha fraterculus* to analyze the presence of histone H3 phosphorylated at positions 10 or 28 (preliminary reported in Giardini et al. 2011). Both variants of histone H3 serve as markers for chromosomal condensation and segregation during mitosis and meiosis (Goto et al. 1999). Using the H3S28ph antibody, we found positive signals in all centromeres (Figure 4), whereas using the H3S10ph antibody, we found characteristic positive signals of chromosome condensation in all the complement, showing the expected behavior of chromosomes during the mitosis (data not shown) (preliminary reported in Giardini et al. 2011). Considering that histone modification patterns are a particularly informative feature in relation to chromatin characterization, our results represent the first epigenetic characterization of *Anastrepha fraterculus* mitotic chromosomes. Although preliminary, these studies allow confirming the acrocentric nature of the autosomes.

## Sex chromosomes in polytene tissues

The existence of polytene chromosomes in the salivary glands of *Anastrepha fraterculus* was first reported by Mendes (1958). Our group characterized these chromosomes and published the first polytene pictures of this species (Giardini et al. 2009a). These chromosomes show homogeneity in chromosome length, similar banding and puffing patterns between sexes, and the absence of a typical chromocentre, resulting in the observation of complete individual chromosomes. We have described each chromosome on the basis of constant morphological structures (landmarks) and specific features (e.g., puffing pattern) and performed an approximation to a linear map following a customary labeling system (see details in Giardini et al. 2009a). Currently, a detailed map of *Anastrepha fraterculus* is in progress (M. Cecilia Giardini, Antigone Zacharopoulou, in preparation).

**Figure 5.**
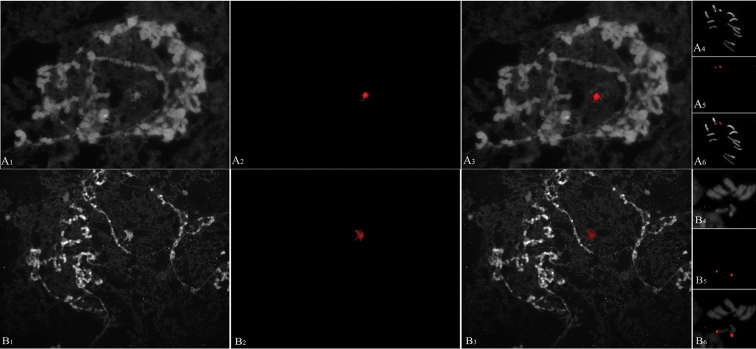
18S rDNA FISH analysis in polytene and mitotic tissues. Polytene and mitotic chromosome preparations obtained from third instar larvae of male (**A**) and female (**B**) of *Anastrepha fraterculus*. In each case: **1** Polytene chromosomes DAPI stain **2** Polytene chromosomes 18S rDNA hybridization signa (FISH) **3** Polytene chromosomes merged image (DAPI/FISH) **4** Mitotic chromosomes DAPI stain **5** Mitotic chromosomes 18S rDNA hybridization signal (FISH) **6** Mitotic chromosomes merged image (DAPI/FISH).

We have also performed a simultaneous analysis of mitotic and polytene nuclei of Argentinean *Anastrepha fraterculus*, and observed that neither the number of polytene chromosomes nor their banding patterns differentiate males from females (Giardini et al. 2009a). This suggests that in *Anastrepha fraterculus*, as well as in other tephritid flies (Zacharopoulou 1987, Mavragani-Tsipidou et al. 1992, Zacharopoulou et al 2011a, 2011b, Garcia-Martinez et al. 2009), the sex chromosomes do not form polytene chromosomes. This finding was tested by FISH experiments using the specific 18S rDNA probe in polytene chromosomes, which revealed a hybridization signal in a region of granular and uncondensed heterochromatin (Figure 5) that corresponded to the non-polytene sex chromosomes (Giardini et al. 2012).

## Conclusion and remarks for the future

**Figure 6.**
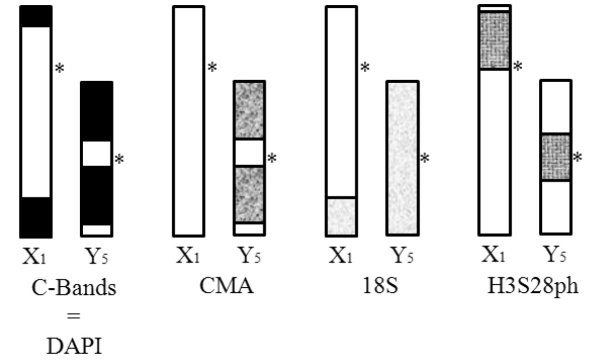
Cytogenetic summary of sexual chromosome pair. Ideogram of sex chromosomes of *Anastrepha fraterculus* from Argentina (most frequent karyotype). Relative location of C-Bands, DAPI/CMA bands, 18S and anti-H3S28ph hybridization signals.

This review summarizes the cytogenetic information available from Argentinean *Anastrepha fraterculus*, focused on sex chromosome variation. Figure 6 shows an ideogram illustrating the results of all the techniques applied so far in the cytogenetic characterization of the sex chromosome pair from mitotic preparations of *Anastrepha fraterculus*. Several structural polymorphisms have been described in sex chromosomes from wild and laboratory Argentinean populations. In contrast to that observed in Brazilian populations, these polymorphisms do not act as reproductive barriers between individuals of different populations. Deeper characterization of the *Anastrepha fraterculus* karyotype by FISH allowed the identification and location of ribosomal genes in terminal position on the sex chromosomes. Chromatin characteristics were also explored, and allowed the specific detection of centromeric regions and chromosomal condensation status in mitotic chromosomes of this species. The first characterization of polytene chromosomes in this species provided the description of landmarks and specific features on this type of chromosomes, and the detection of sex chromosomes as granular and uncondensed heterochromatin in polytene tissues.

All the results described here represent valuable information to be further used in the identification of genetic entities in the *Anastrepha fraterculus* complex of cryptic species. Deeper characterization of the structural variation of the sex chromosomes and polytene chromosome needs to be addressed to have a complete genetic picture of this species, which represents one of the most destructive fruit flies of economic importance in Argentina and the South American region. A detailed taxonomic revision of *Anastrepha fraterculus* and the accurate elucidation of the complexity displayed by this species in South America are of uttermost importance to develop environment-friendly autocidal control methods as is the Sterile Insect Technique (SIT), ensuring its specificity and effectiveness.
